# Associations of lesion location, structural disconnection, and functional diaschisis with depressive symptoms post stroke

**DOI:** 10.3389/fneur.2023.1144228

**Published:** 2023-05-17

**Authors:** Julian Klingbeil, Max-Lennart Brandt, Anika Stockert, Petra Baum, Karl-Titus Hoffmann, Dorothee Saur, Max Wawrzyniak

**Affiliations:** ^1^Neuroimaging Laboratory, Department of Neurology, University of Leipzig Medical Center, Leipzig, Germany; ^2^Department of Neurology, University of Leipzig Medical Center, Leipzig, Germany; ^3^Department of Neuroradiology, University of Leipzig Medical Center, Leipzig, Germany

**Keywords:** stroke, depression, diaschisis, disconnection, lesion network mapping

## Abstract

**Introduction:**

Post-stroke depressive symptoms (PSDS) are common and relevant for patient outcome, but their complex pathophysiology is ill understood. It likely involves social, psychological and biological factors. Lesion location is a readily available information in stroke patients, but it is unclear if the neurobiological substrates of PSDS are spatially localized. Building on previous analyses, we sought to determine if PSDS are associated with specific lesion locations, structural disconnection and/or localized functional diaschisis.

**Methods:**

In a prospective observational study, we examined 270 patients with first-ever stroke with the Hospital Anxiety and Depression Scale (HADS) around 6 months post-stroke. Based on individual lesion locations and the depression subscale of the HADS we performed support vector regression lesion-symptom mapping, structural-disconnection-symptom mapping and functional lesion network-symptom-mapping, in a reanalysis of this previously published cohort to infer structure–function relationships.

**Results:**

We found that depressive symptoms were associated with (i) lesions in the right insula, right putamen, inferior frontal gyrus and right amygdala and (ii) structural disconnection in the right temporal lobe. In contrast, we found no association with localized functional diaschisis. In addition, we were unable to confirm a previously described association between depressive symptom load and a network damage score derived from functional disconnection maps.

**Discussion:**

Based on our results, and other recent lesion studies, we see growing evidence for a prominent role of right frontostriatal brain circuits in PSDS.

## 1. Introduction

Post-stroke depressive symptoms (PSDS) impose a significant burden on stroke survivors and are independent predictors of worse functional outcome and increased mortality (1, 2). From a clinical perspective, it is important to identify patients at risk of post-stroke depression (PSD) early on to provide adequate treatment and ensure optimal rehabilitation despite the depressive symptoms. Risk prediction based on medical factors and psychiatric history could promote timely screening for and recognition of depressive symptoms (3). In addition, lesion location is a readily available information in stroke patients and might be useful to estimate the individual risk for PSD (4). The assumption that the biological effect of a stroke lesion may also contribute to PSD is consistent with the biopsychosocial disease model of major depressive disorder (MDD) (5). Mounting evidence indicates that PSD is not just caused by psychosocial factors such as difficulties to adjust to the new physical disabilities or the suddenly altered living circumstances, but could also be a direct consequence of brain damage (6). Strong support for this conclusion comes from a recent, large study that identified a 50% higher risk to develop a depressive disorder up to 1.5 years after the event when comparing stroke to myocardial infarction (7). But unlike MDD, PSD offers the possibility to draw causal inferences on the neural substrates of depressive symptoms based on lesion locations (6). Structure–function inference with stroke lesions has contributed significantly to our understanding of the human brain in the past two centuries (8). Admittedly, studies on lesion location and mood have had mixed results: despite over 80 lesion studies, no consistent association has been identified (9). But recently, larger studies using modern methods of structure–function inference such as voxel-based lesion-symptom mapping (VLSM) showed promising results (10–12).

Yet, inference for PSDS based on direct lesion effects may also fail despite the methodological advances of lesion-symptom mapping (4, 6, 13), because complex brain functions such as language or mood arise from interactions between large-scale distributed networks rather than single or specific brain regions (8). Lesions that fall into such networks are thought to also cause symptoms due to functional diaschisis (14, 15) or structural disconnection (16–18). The identification of diaschisis and disconnection in distributed networks as a cause of PSDS requires different methodological approaches. Several new methods have become available in the last decade. Functional diaschisis and structural disconnection can for example be examined based on normative functional and structural connectome data (8, 16, 17, 19). Three recent studies on PSDS applied these methods. Weaver and colleagues used measures of structural disconnection and identified right frontal cortico-striatal-thalamic circuits to be associated with PSDS (11). With a similar method, Pan and colleagues identified an association of structural disconnection with PSD bilaterally in the temporal, prefrontal and parietal white matter and the posterior corpus callosum (12). Furthermore, the inclusion of such indirect measures of structural disconnection improved predictive models for PSD (12). Padmanabhan and colleagues, on the other hand, used indirect measures of functional diaschisis to predict depressive symptoms (4). They demonstrated that the overlap between lesions and a depression circuit derived from the functional connectivity of the left DLPFC correlated with PSDS (4).

Here, we provide a reanalysis of a recently published large sample of stroke patients evaluated for depressive symptoms 6 months after stroke. In our recent study, we demonstrated an association of the right basal ganglia with PSDS using VLSM in the sense of a mass-univariate approach (10). In extension to that study, we here used multivariate analyses in combination with methods based on lesion location, structural disconnection and functional diaschisis to identify regions where lesions, disconnection or functional diaschisis might cause the development of depressive symptoms.

## 2. Methods

### 2.1. Patient recruitment and behavioral testing

Institutional review boards approved all study protocols and informed consent for study participation was obtained from all participants (or their legally designated surrogates). First ever stroke patients were recruited from the stroke unit of the Department of Neurology, University of Leipzig Medical Center from 01/2012 to 12/2014 and 11/2017 to 11/2018 as previously described (10). We excluded patients not speaking German, with a history of depression, other psychiatric or neurologic disorders affecting the CNS or other severe diseases and patients aged < 18 or > 90 years. For 270 patients, behavioral scores from the Hospital Anxiety and Depression Scale (HADS) around 6 months after stroke (189.5 ± 10.3 days, range 159–284) were available (10). We used the depression subscale (HADS-D) as a continuous measure for the severity of depressive symptoms in the subsequent analyses. However, to make our results comparable to those of Padmanabhan et al., we also used a cut-off value of > 10 on the HADS-D for the lesion network-symptom-mapping analyses. Stroke-related disability was quantified with the National Institute of Health Stroke Scale (NIHSS) and the Barthel-Index in the first weeks after stroke as previously described (10).

### 2.2. Brain imaging and preprocessing

We used pseudonymized clinical imaging acquired during clinical routine examinations at the Department of Neuroradiology, University of Leipzig Medical Center with the imaging and preprocessing procedures previously described (10). In brief, lesions were first delineated by two reviewers blinded to the patients’ outcome in native space on 202 MRIs and 68 CTs with the semi-automated Clusterize Toolbox (20), manually edited using MRIcron (21) and finally supervised by a neurologist experienced in neuroimaging (JK). These lesions were used for cost-function masking during normalization of the corresponding MRI and CT scans to MNI (Montreal Neurological Institute) space. For spatial normalization, we used the Clinical Toolbox (22) for SPM12 (Wellcome Trust Centre for Neuroimaging, London, UK, RRID:SCR_007037) running on MATLAB (R2019a, The MathWorks Inc., Natick, MA, RRID:SCR_001622) and resliced all images to 1 mm isotropic voxels. The resulting non-linear normalization parameters were also applied to the native space lesion maps, which were then used for further analyses in MNI space.

### 2.3. SVR-LSM

In contrast to the univariate approach used in our previous publication (10), we here used multivariate support vector regression lesion-symptom mapping (SVR-LSM) to infer direct lesion-symptom relationships. We resampled all lesion maps to 2 mm isotropic voxels to enable reasonable computing times. All SVR-LSM analyses were performed with version 2 the multivariate lesion symptom mapping toolbox of DeMarco and Turkeltaub (23). Only voxels damaged in ≥ 5 patients were included, which resulted in the exclusion of 13 out of 270 lesions because they had no voxels inside the minimum lesion cutoff mask implemented in the toolbox. Lesion volume was controlled for in all analyses by regressing it out from both the behavioral data (HADS-D) and the raw lesion data (23). We used default values for the hyperparameters (see Supplementary Table S3) (23, 24). Prediction performance was calculated in-sample by determining the mean Pearson correlation coefficient between the real and predicted depression scores and ranked relative to 5,000 permuted models (see Supplementary Table S3). Statistical inference was based on SVR β-maps thresholded using the null-distribution of cluster sizes obtained by 5,000 random permutations with a threshold of *p* < 0.005 (uncorrected, one-tailed) on the voxel-level and of p(FWE) < 0.05 on the cluster-level. The analyses were repeated with age, sex, stroke severity (NIHSS) and functional impairment (Barthel-Index) as additional covariates, in analogy to the analyses described by Weaver and colleagues (11). To this end, the covariates were regressed out of behavior of interest (HADS-D) prior to the SVR-LSM. Notably, our analysis with covariates differed in two important aspects from the analysis by Weaver and colleagues – the measures where only available from the acute phase after stroke (6.1 ± 3.5 d post-stroke) and no measure for cognitive deficits was collected.

### 2.4. SVR-SDSM

We used a combination of support vector regression and structural disconnection mapping (support vector regression structural disconnection-symptom mapping, SVR-SDSM) to infer relationships between structural disconnection and depressive symptoms. Structural disconnection mapping was performed with BCBtoolkit (16). Deterministic fiber tracking seeding from the individual lesion masks was performed in the 10 healthy participants provided with the toolkit and transformed to MNI space. The resulting maps were binarized and overlapped for each patient resulting in individual disconnectome maps with values between 0 and 100%. These disconnectome maps were again binarized with a cutoff of ≥ 60%, since this cutoff had been shown to be optimal in a systematic evaluation of the method (25). Relationships between the binary disconnectome maps and depressive symptoms were analyzed in analogy to SVR-LSM described above. Five disconnectome maps were excluded because they had no voxels inside the minimum lesion mask. In all five cases, the corresponding lesions were very small cortical lesions for which the fiber-tracking algorithm failed to generate a meaningful disconnectome map. For the multivariate analyses, again, the multivariate lesion symptom mapping toolbox was used as described above (23). Instead of lesion maps, the binarized structural disconnection maps were used. All structural disconnection maps were resampled to 2 mm isotropic voxels prior to SVR-SDSM.

### 2.5. LNSM

Lesion network-symptom mapping (LNSM) was used to infer relationships between functional diaschisis and symptoms. The concept behind this method is that regions functionally connected to the lesion site are vulnerable to diaschisis effects. These analyses were performed with SPM12 and in-house tools with MATLAB as previously described (26, 27). LNSM was performed using a mass-univariate approach to avoid binarizing the functional map using arbitrary thresholds (26, 28). Specifically, we used functional connectome data (*n* = 100, young unrelated healthy adults) from the human connectome project (29). The functional data sets included two 15 min resting-state sessions (right–left and left–right phase encoding) with gradient-echo EPI sequence (TR of 720 ms, 2 mm isotropic voxels) and were downloaded already ‘minimally preprocessed’ (gradient distortion correction, motion correction, distortion correction, normalization to MNI space, intensity normalization and bias field removal) (30). We convolved all functional images with an isotropic Gaussian smoothing kernel (FWHM = 5 mm). Signal variance over time explained by nuisance variables (motion parameters, mean white matter, CSF and global signal) was removed using multiple regression. Residual BOLD time series were band-pass filtered (0.01–0.08 Hz). All images with a frame-wise displacement > 0.5 mm were discarded (31). Additionally, two data sets with heavy in-scanner motion were excluded entirely. Individual lesion masks were the regions of interest (ROIs) from which representative BOLD time series were extracted as the first eigenvariate of the time series of all voxels within that ROI. These ROIs were defined exclusively as the gray matter portion of the individual lesion masks, as meaningful BOLD signal is restricted to the gray matter (32, 33). This was achieved by masking the lesions with the gray matter probability mask provided with the functional data thresholded at 10% and resulted in the exclusion of 14 patients with pure white matter lesions. Finally, lesion networks were calculated based on functional connectivity (i.e., Fisher-transformed Pearson correlation coefficients) between the ROI time series and the time series of all other brain voxels. All connectivity maps (from 98 controls, separate for right–left and left–right phase encoding) were averaged to obtain a single functional lesion network map for every lesion. These individual lesion network maps represent regions potentially affected by functional diaschisis. LNSM was then carried out as described before with non-parametric permutation testing (26, 34). Continuous scores from the HADS-D were entered into a regression analysis using a mass-univariate general linear model. The statistical inference was based on the null-distribution of cluster sizes (using a voxelwise threshold of *p* < 0.001) obtained with 5,000 random permutations. The result was thresholded at p(FWE) < 0.05. All analyses were restricted to voxels with at least 10% gray matter tissue probability. In analogy to Padmanabhan et al. (4), the analyses were repeated in a mask for the middle frontal gyrus (MFG) derived from the Harvard-Oxford atlases (35) without gray matter masking, with a correction p(FWE) < 0.05 on cluster- and voxel-level, without correction for multiple comparisons and with a cut-off value of HADS-D > 10 for PSD. For the calculation of a network damage score which might be predictive for PSDS, we also followed the procedure described by Padmanabhan et al. (4). Since all analyses using a MFG mask were negative, we chose a spherical ROI with a 9 mm diameter around the peak coordinates (MNI: x = −32, y = 12, z = 36) reported by Padmanabhan and colleagues (4). The network damage score was calculated as follows: a network map for this ROI, representing the ‘depression circuit’, was computed using the normative functional connectome data as described above. Then for each of our patients a network damage score was computed as the sum of the intensity (*t*-values) of all voxels in the depression circuit that overlap with the patient’s lesion. Lesion size was controlled with a residualized network damage score after regression against lesion size. Then these network damage scores were compared between patients with depression (HADS-D > 10) and those without (HADS-D < 11). Statistical significance was calculated using a permutation equivalent of a t-test with one million permutations (4). The analyses were repeated with a HADS-D of > 7 as a cut-off and continuous HADS-D values.

## 3. Results

**Figure 1:**
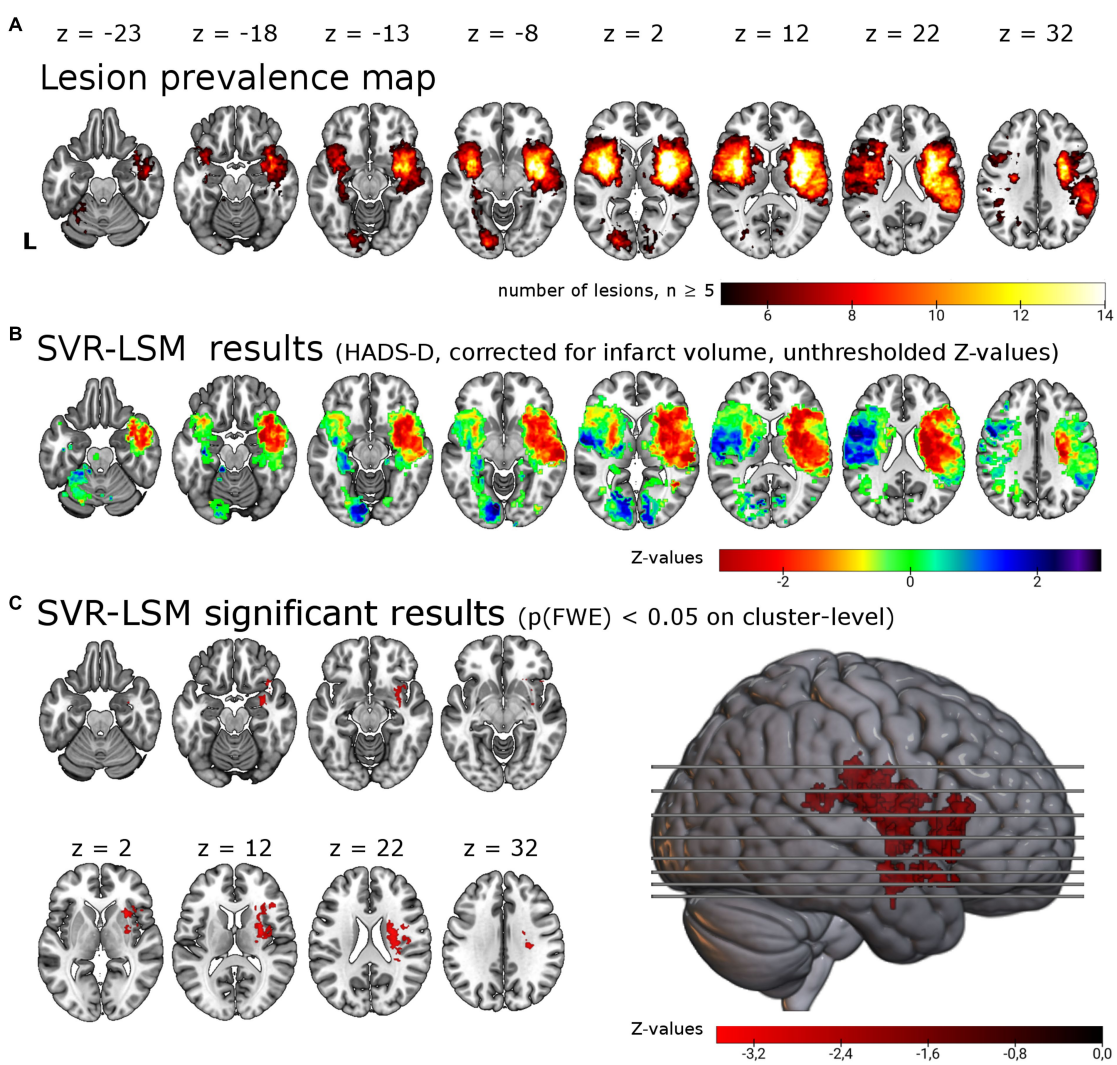
Support vector regression lesion-symptom mapping for depressive symptoms 6 months post-stroke. **(A)** Lesion overlap of all 257 patients included in the analysis masked with a minimum lesion overlap of ≥ 5. **(B)** Unthresholded results for the SVR-LSM with the continuous HADS-D scale. Note that values (*z*-scores) < 0 in warm colors correspond to an association between lesions and higher symptom scores. **(C)** Results thresholded with *p* < 0.005 on voxel-level and p(FWE) < 0.05 on cluster-level resulted in a single cluster of 12.49 ml in the right hemisphere, shown here in the lateral view and axial slices. The cluster encompasses in particular the right insula (22.0% of the cluster), the right putamen (18.1%) and the right inferior frontal gyrus (14.2%). L = left.

Mean HADS-D 6 months post stroke was 4.4 ± 3.7. Lesions were rather small (18.3 ± 38.4 ml) with a predominantly subcortical distribution (see Figure 1A). In patients with HADS-D > 7, lesions were larger (34.0 vs. 14.5 ml, *p* < 0.01). Median time post-stroke for the imaging used to delineate lesions was 5 days (interquartile range 4 days). Further clinical and demographic characteristics are provided in Supplementary Table S1.

### 3.1. SVR-LSM

We tested for associations of lesion location with depressive symptoms post-stroke using lesion locations and the HADS-D 6 months post-stroke in a multivariate lesion symptom mapping approach (SVR-LSM). Lesion overlap with a minimum of 5 lesions covered 20.0% of the brain mask, thus most frontal, parietal, temporal, occipital, cerebellar and brain stem regions were not included in the analysis (see lesion overlap in Figure 1A). With SVR-LSM, one cluster of 12.49 ml [p(FWE) = 0.004] in the right hemisphere survived permutation-based FWE-correction on cluster-level. This cluster localized mainly to the subcortical white matter and the putaminal, insular and inferior frontal gray matter (see Figure 1C; Supplementary Table S2). The white matter tracts with most overlap where the right corticospinal tract and the superior thalamic radiation and to a lesser extent the right inferior fronto-occipital fasciculus [labels based on the XTRACT atlas (36)]. The subcortical gray matter with most overlap was the right putamen and to a lesser extent the caudate nucleus. The main cortical gray matter structures with most overlap were the right insular cortex and the right inferior frontal gyrus and to a lesser extent the right superior, middle and inferior temporal gyrus, the right orbitofrontal cortex, the right hippocampus [based on the LONI atlas (37)] and the right amygdala [based on the Harvard-Oxford brain atlas (35)]. When the analyses were repeated with age, sex, stroke severity (NIHSS) and functional impairment (Barthel-Index) as covariate in analogy with the analyses by Weaver and colleagues, the identified cluster was smaller but with a similar anatomical distribution [one cluster in the right hemisphere with 6.4 ml, p(FWE) = 0.023, see Supplementary Table S2].

### 3.2. SVR-SDSM

**Figure 2:**
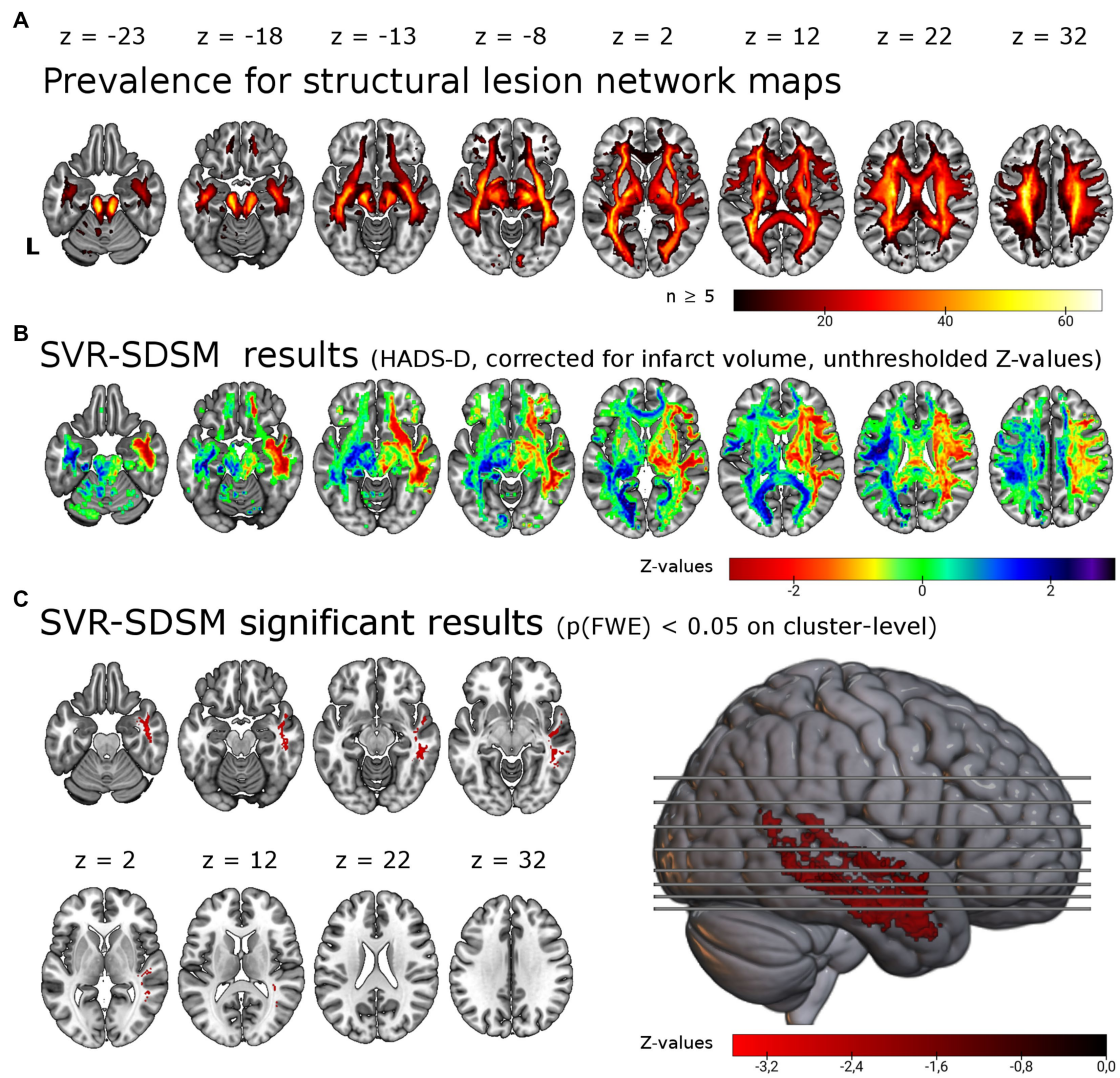
Support vector regression structural-disconnection mapping for depressive symptoms 6 months post-stroke. **(A)** Overlap of all binarized (threshold of ≥ 60%) disconnection maps shows good coverage of cerebral white matter. The map is restricted to a minimum overlap of ≥ 5 disconnection maps. **(B)** Unthresholded results for the SVR-SDSM with the continuous HADS-D score. Note that values (*z*-scores) < 0 in warm colors correspond to an association of disconnection with higher symptom scores. **(C)** Results thresholded with *p* < 0.005 on voxel-level and p(FWE) < 0.05 on cluster-level resulted in a single cluster in the right temporal lobe, shown here in the lateral view and axial slices. This significant cluster (*p* = 0.024) of 5.86 ml size encompasses in particular the right inferior longitudinal fasciculus (37.7% of the cluster), the right middle longitudinal fasciculus (20.6%) and right the uncinate fasciculus (8.6%). L = left.

We tested for associations of structural disconnection with depressive symptoms post-stroke using lesion derived structural connectivity and the HADS-D 6 months post-stroke in a multivariate lesion symptom mapping approach (SVR-SDSM). As opposed to the analyses of lesion locations (SVR-LSM), structural disconnection maps seeded from the individual lesion masks were the basis for this set of analyses. Overlap of the binarized structural disconnection maps with a minimum of five resulted in the inclusion of most supratentorial white matter tracts and the corticospinal tract (see Figures 2A,B). With SVR-SDSM one cluster of 5.86 ml in the right hemisphere survived permutation-based FWE-correction on cluster-level with p(FWE) = 0.024. This cluster was localized in the white matter of the right temporal lobe (see Figure 2C; Supplementary Table S2) beneath the inferior, middle and superior temporal gyrus [based on the LONI atlas (37)]. The white matter tracts overlapping with this cluster were the right inferior longitudinal fasciculus, the right middle longitudinal fasciculus and the uncinate fasciculus [with labels based on the XTRACT atlas (36)]. The second largest cluster, which was not significant [p(FWE) = 0.108, 1.67 ml], was located in the right frontal lobe mostly within the uncinate fasciculus. Disconnection in these regions was associated with more severe depressive symptoms but there were no voxels where disconnection was associated with a lower depressive symptom score.

### 3.3. LNSM

**Figure 3:**
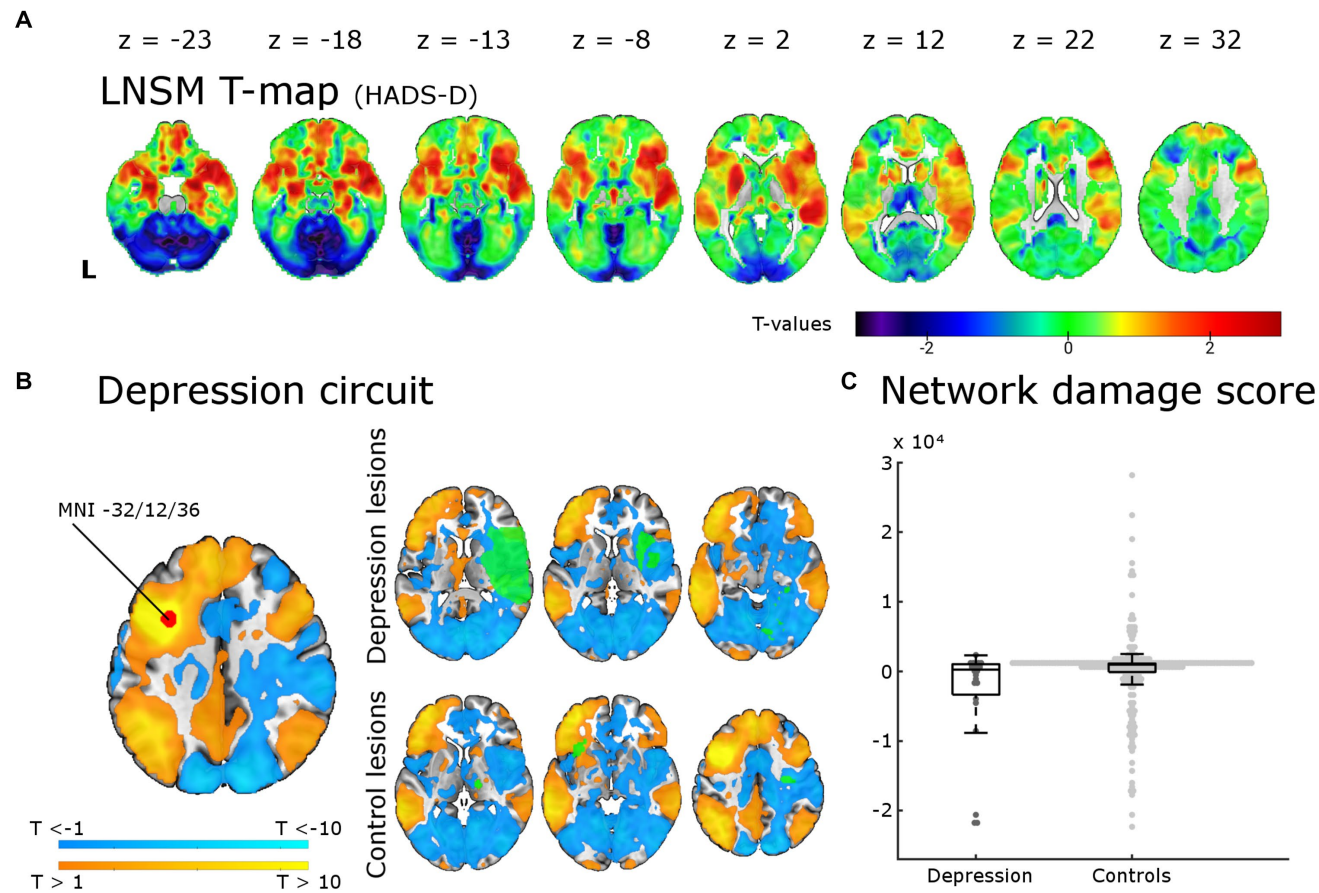
Lesion network-symptom mapping for depressive symptoms 6 months post-stroke and network damage scores. Results from the LNSM with the continuous HADS-D scale at 6 months post-stroke. **(A)** The unthresholded map of T-values shows a bilateral frontal, temporal and basal ganglia maximum, but there was no significant association between functional lesion network map strength and depressive symptoms [p(FWE) < 0.05 at cluster-level]. The analysis remained negative also when applying a mask for the bilateral middle frontal gyrus, when using a binary cut-off at HADS-D > 10 and when uncorrected for multiple comparisons (not shown here). **(B)** The depression circuit was derived from a region-of-interest (ROI) with a 9 mm diameter sphere (shown in red) around the peak coordinates (*x* = −32, *y* = 12, *z* = 36) reported by Padmanabhan and colleagues. The ROIs whole brain functional connectivity was calculated with the normative connectome of 100 healthy subjects. Warmer colors indicating positive connectivity to the ROI and cool colors negative connectivity. This constitutes the ‘depression circuit’ as described by Padmanabhan and colleagues. In green six random lesions of patients with and without depressive symptoms (binary cut-off > 10 on the HADS-D) are shown for illustration as an overlay on the ‘depression circuit’ – the intersection of the lesions with the depression circuit then results in the network damage score. **(C)** The network damage scores of patients with and without severe depressive symptoms did not differ significantly (*p* = 0.93). All data points are shown in the box plot with the exception of one outlier (NDS 6.3 × 10^4^, depression group).

We tested for associations of regions potentially affected by functional diaschisis with depressive symptoms post-stroke using lesion derived functional connectivity and the HADS-D 6 months post-stroke in a mass-univariate approach. Figure 3A displays unthresholded LNSM results. We found no statistically significant association between functional lesion network strength and depressive symptoms post-stroke. Following the results of Padmanabhan et al. in a second step, we restricted the analysis to the left and right MFG. This also resulted in no significant association: regardless of the use or omission of gray matter masking, of cluster-or voxel-level inference and even the very liberal significance threshold p(uncorrected) < 0.05 of Padmanabhan et al. (4). Finally, we calculated the ‘depression circuit’ (see Figure 3B) in analogy to Padmanabhan et al. (4). Patients with more severe depressive symptoms (HADS-D > 10) did not differ from those with less depressive symptoms (*p* = 0.93) in their network damage scores (see Figure 3C). This also remained unchanged with different cut-offs (HADS-D > 7: *p* = 0.57; continuous HADS-D: *p* = 0.48).

## 4. Discussion

We combined three recent approaches to infer structure–function relationships in this lesion-symptom mapping study for post-stroke depressive symptoms in a large patient cohort. We analyzed relationships between PSDS 6 months after stroke and lesion location (SVR-LSM), structural disconnection (SVR-SDSM) and localized functional diaschisis (LNSM). We identified an association of higher depression scores with (1) lesions in the right insular cortex, putamen and inferior frontal gyrus and (2) structural disconnection in the white matter of the right temporal lobe, but (3) no association with localized functional diaschisis.

The direct effects of lesion locations on depressive symptoms have been extensively studied in PSD over the past 40 years, yet results on specific brain regions, anterior–posterior gradients or even lesion laterality are too heterogeneous to draw consistent conclusions (6, 9). This has been ascribed, in part, to the rather imprecise methods used for structure–function inference, but may be overcome with voxel-based methods (38). With SVR-LSM, we identified an association of PSDS with lesions in several cortical and subcortical gray matter regions and the subcortical white matter. The structures affected to the largest extend were the right insula, the right putamen and the right inferior frontal gyrus along with the right corticospinal tract and the right superior thalamic radiation. This replicates and extends our previous finding using univariate VLSM, were we identified a significant association of PSDS with lesions of the right dorsal putamen (10). However, all eight present VLSM studies together are not conclusive (6): three studies were based on small samples in specific brain regions (39–41) and two larger studies were negative (4, 13). But there are now three lesion-symptom-mapping studies – with a total of nearly 2,000 patients – that identify an association of post-stroke depressive symptoms with specific lesion locations: the study by Weaver and colleagues (11), the recent study by Pan and colleagues (6) and the current analysis. The results are at least partly overlapping: the associated lesion locations are mainly (11) or exclusively [(12), this study] located in the right hemisphere, affect both white matter tracts and gray matter regions [(11, 12), this study] and encompass both basal ganglia [(11), this study] and cortical gray matter [(11, 12), this study]. The study by Weaver and colleagues represented a significant advance because of the multivariate LSM approach (11, 23, 24). By analyzing our independent data in a very similar way, we are able to confirm several of their findings. Namely the involvement of the right basal ganglia, the right hippocampus and right amygdala. Since our prior mass-univariate analysis only identified the right putamen (10), this reanalysis likely demonstrates a higher sensitivity of multivariate approaches to unveil the neurobiological basis of more complex brain functions represented in distributed brain networks and therefore hidden to classic VLSM analyses (6, 23, 24, 38). Notably, the results of Weaver and colleagues and our cohort converge in the right basal ganglia and amygdala despite differences in the time point (3 vs. 6 months), behavioral assessment (GDS vs. HADS) and patient cohort (Korean vs. German) (11). A meta-analysis of previous studies also identified an association of right hemispheric lesions with depressive symptoms 1–6 months post stroke (42). Weaver and colleagues and Pan and colleagues were reluctant to draw the conclusion that the right hemisphere is specifically associated with PSDS (6, 43). It is certainly true that these results implicate several regions in the right hemisphere with PSDS rather than the right hemisphere itself (11). Moreover, since absence of evidence is not evidence of absence, we cannot dismiss a possible contribution of regions within the left hemisphere based on our results. It is, for instance, possible that the left basal ganglia may also be involved (see Figure 1A), but were not significant due to insufficient statistical power. Still, one may conclude that it is at least unlikely that damage in almost all left cortical regions covered by our analyses contributes to PSDS (see direction of effect in the left cortical regions in Figure 1A). But the area with sufficient lesion overlap for the analyses was small, so only about 20% the brain was included in these analyses (see Figure 1B). The underrepresentation of patients with moderate to severe aphasia and thus large cortical lesions in the left frontal and temporal lobe certainly contributed to the limited lesion coverage and precludes the inference of a right-lateralized depression network. Since we used the HADS, patients with moderate to severe language comprehension deficits could not be included. With the Aphasic Depression Rating Scale, a possible alternative is available (44). A large patient cohort assessed with this scale and/or a psychiatric examination could provide a spatially less biased analysis.

We believe that the diverse regions identified must be understood in a connectome based account of depression as a network disorder (8, 45). Several of the identified regions are plausible major components of such a depression network. The subcortical gray matter regions identified were the right striatum [(10, 11), this analysis] and the right pallidum (11). The striatum has been consistently implied in MDD and PSD due to its prominent role in reward mechanisms, anhedonia, apathy and motivation (46, 47). Both accelerated striatal gray matter volume loss in MDD (48) and prediction of MDD based on lower striatal volume have been reported (49). Moreover, depression is highly prevalent in Parkinson’s disease, which is in turn characterized by striatal dysfunction (50, 51). Also, deep brain stimulation of the striatum (ventral putamen) and nucleus accumbens is effective for the treatment of MDD (52, 53). The cortical regions involved are more diverse. The prefrontal cortex is the cortical region most consistently associated with MDD (54) and the left dorsolateral prefrontal cortex has been established as the most reliable site for the transcranial magnetic stimulation treatment of MDD (55). None of the three large lesion-symptom mapping studies identified the dorsolateral prefrontal cortex, but several other areas of gray matter: inferior frontal gyrus [(12), this analysis], insula [(12), this analysis], superior and middle temporal gyrus [(12), this analysis], inferior temporal gyrus (11), inferior parietal cortex (12), and the amygdala and hippocampus [(11), this analysis]. The anterior insula is one of the functionally most diverse structures in the brain, but it has been consistently associated with emotion regulation (56) and has even been discussed as the location where subjective feelings of emotion are generated (57, 58). Reduced gray matter volume in the right insula, among many other regions, has been conclusively demonstrated in MDD (59). The amygdala is a central part of the emotion circuits of the brain (60) and reduced gray matter volume in the amygdala has been described in late life depression (61) and MDD (62, 63). The right inferior frontal gyrus has also been implied in MDD in the sense that larger gray matter volume in the IFG predicted better clinical outcome in MDD after 5 years (54), both striatum and IFG are relevant for reward mechanisms (64) and reduced IFG activation leads to negative processing bias (65). Several white matter regions were also consistently associated with PSDS in lesion-symptom mapping analyses: the corona radiata [(11, 12), this analysis], the superior longitudinal fasciculus (11, 12) and the posterior thalamic radiation (11, 12). Increased fractional anisotropy has been described in the superior longitudinal fasciculus and the internal capsule in MDD compared to healthy controls (66).

Together, the identification of the right frontal operculum and the right putamen support a frontostriatal model of PSD (6). Frontostriatal dysfunction has been identified both in MDD and late life depression (67). Clinically, patients with late life depression, but also MDD, are characterized by prominent dysexecutive symptoms together with depressed mood. In these patients the disproportionate affection of frontostriatal connections has been proposed to be the neurobiological correlate of depressed mood and executive dysfunction (67). Cognitive deficits are also predictive of PSD (1). Further evidence for a frontostriatal theory of depression comes from neuromodulatory therapeutic interventions in MDD: both deep brain stimulation in the ventral capsule/ventral striatum and transcranial magnetic stimulation in the dorsolateral prefrontal cortex are effective (53).

In addition to the direct lesion effects discussed up to this point, the depression network may also become dysfunctional when its nodes are structurally disconnected or affected by functional diaschisis. Because both, white and gray matter seem to be involved in PSDS, we complemented our analyses of direct lesion effects (SVR-LSM) with indirect methods in search of both structural disconnection (SVR-SDSM) and functional diaschisis (LNSM, network damage score). We identified an association of PSDS with structural disconnection in the white matter of the right temporal lobe in our cohort, but could not find an association with localized functional diaschisis.

Affection of the uncinate fasciculus has been implied in MDD (68) and dysfunction of the superior longitudinal fasciculus in rumination (69) and suicidal ideation (70). An association between white matter damage and depressive symptoms has long been assumed based on the studies of white matter hyperintensities in late life ‘vascular depression’ (71). The disproportionate decline of white matter compared to gray matter due to cerebral small vessel disease led to the disconnection hypothesis of vascular depression (67). The best evidence for a contribution of structural disconnection in white matter tracts that pass through the temporal lobes comes from studies on white matter hyperintensities and late-life depression (72). Structural disconnection in the right temporal lobe in patients with PSDS was also identified by two other studies that used indirect measures of structural disconnection (11, 12). Apart from the right parahippocampal white matter, they identified the right anterior thalamic radiation (11) and bilateral temporal white matter, bilateral prefrontal and posterior parietal white matter and the posterior corpus callosum (12). Taken together, the three studies that used indirect measures of structural disconnection demonstrate partially overlapping results with the best evidence for an involvement of the right temporal white matter. In our cohort, we found no association of PSDS with structural disconnection in the left hemisphere (see Figure 2A). In contrast the study by Pan and colleagues provides strong evidence for a bilateral pattern of structural disconnection (12). Based on their large and well-characterized cohort they were able to go further and calculate a structural damage score. The score was derived from the degree of overlap of individual disconnection maps with the white matter regions where an association of structural diaschisis with PSD had been identified. This structural disconnection score was the strongest predictor in a multifactorial prediction model that included known risk factors of PSD such as cognitive deficits, stroke severity, functional status, sex, lesion size, and age (12). Taken together, the three studies based on indirect measures for structural diaschisis support a brain network theory of depression and point to a prominent role of frontal and temporal structural disconnection underlying PSDS. The study by Pan and colleagues furthermore provides evidence for the behavioral relevance of structural disconnection in PSD and its possible application in multifactorial prediction models for PSD (6).

We were unable to reproduce the results reported by Padmanabhan et al. who had identified functional diaschisis in the DPLFC in PSD patients and described a ‘depression circuit’ that had the potential to predict PSD based on lesion location. The strongest support for an involvement of the DLPFC in patients with MDD comes from studies converging on the left DLPFC as a suitable target for a network-guided transcranial magnetic stimulation treatment of depressive symptoms (73, 74). This has even been demonstrated in patients with PSD (75, 76). The lack of consistency of our result with these findings might be explained by differences in lesion aetiology (only stroke vs. stroke, intracerebral haemorrhage and traumatic brain lesion), behavioral scales (HADS scale vs. several other scales) or the time of assessment (6 months vs. 3 months – 30 years post-stroke). Alternatively it could be related to the method itself: while LNSM has certainly contributed to the understanding of the network damage in several neurological and psychiatric symptoms and syndromes [e.g., (19, 25, 26, 77–81)], it has also been demonstrated that indirect measures of functional diaschisis – such as LNSM – explain less variance in stroke symptoms than indirect measures of structural connectivity, direct measures of functional connectivity and lesion location (82, 83). Moreover, functional lesion network-mapping tends to generate anatomically plausible patterns, which is also the case here (see Figure 1A where many of the regions identified with the other two methods seem to fall onto the functional disconnection map), but accounts for very little behavioral variance (82). The direct measurement of functional diaschisis, although laborious in acute stroke patients, has been proposed to better understand the networks involved and discover compensatory mechanism that may be exploited therapeutically (6, 82). Methodological improvements of LNM may also prove fruitful. A recent work by Trapp and colleagues, which was conceptually similar to the LNM analysis presented here but used a different analytic approach, did indeed show that diaschisis to specific brain regions has an association with higher or lower risk for developing depressive symptoms. To uncover this association they had to rely on a very large cohort of >500 patients with different lesion aetiologies because of the at best modest strength of the uncovered correlation. They demonstrate that the ‘risk’ and ‘resilience’ regions “are not randomly distributed but fall primarily within two functional networks with lesions of the salience network associated with increased depressive symptoms (‘risk’ nodes) and lesions of default mode network associated with reduced depressive symptoms (‘resilience’ nodes)” (84). Thus, indirect measures of diaschisis might indeed be useful for the prediction of PSDS, but only as one additional factor in a multifactorial biopsychosocial disease model for PSDS. Their work and a study by Pini et al. show that further methodological adaptations of LNM can improve structure–function inference (85). But even if LNM may not be useful for prediction, the accurate anatomical distribution of functional connectivity maps may still be used to determine the regions involved (53). With this approach, Siddiqi and colleagues have described the convergence of stroke lesions that cause depression (also evaluated in a large cohort of 461 patients) and stimulation sites used to treat depression (with deep brain or transcranial magnetic stimulation) on a common brain circuit. This circuit is characterized by positive functional connectivity bilaterally to the dorsolateral prefrontal cortex, frontal eye fields, inferior frontal gyrus, intraparietal sulcus and extrastriate visual cortex and negative connectivity to the subgenual cingulate cortex and ventromedial prefrontal cortex, thus implying even more regions bilaterally in a distributed depression network (53). In addition and in an unexpected twist, they have recently demonstrated that LNM of white matter lesions in multiple sclerosis patients with depressive symptoms maps onto the same depression network (86).

We believe that our analyses are not conclusive because of several further limitations. An important limitation is the small area with sufficient lesion coverage for SVR-VLSM (see Figure 1B). Our study shares this limitation with many previous VLSM studies (38, 87). It has recently been estimated that sample sizes of up to 3,000 patients are needed to achieve a sufficient lesion coverage for true whole brain analyses (87), although it must be added that very rarely affected brain regions can contribute little variance to frequent symptom such as PSD. Data sharing can overcome insufficient lesion coverage. While our data may not be made publicly available due to data protection regulations, we have and will share them upon request. Second, HADS is an established tool to screen for depression but not suited to establish the diagnosis and is probably not the most sensitive and specific screening instrument (88). Since depression is not a uniform phenomenon, a more detailed behavioral characterization might prove fruitful to differentiate lesion effects on depressive subsymptoms (i.e., anhedonia or cognitive control) which are likely to arise from dysfunction in different neuronal circuits. Even more so since depression may itself be understood, in the psychopathological network theory, as a complex network of symptoms where symptom-symptom interactions drive and sustain the depressive symptoms (6, 89, 90). If so, structure–function inference could be improved with the identification of driving symptoms which should then be related to lesion locations, disconnection or diaschisis (6). Third, we have no measures on cognitive impairments or functional impairment after 6 months and thus cannot account for their potential confounding effect. Fourth, in our cohort lesion size correlated with depressive symptoms (see Supplementary Table S1). While we controlled for lesion size in SVR-LSM and SVR-SDSM as recommended, we cannot completely rule out an effect of lesion size on our results since the effect of lesion size is not spatially homogenous (23, 91). Fifth, because of the cluster based inference it cannot be concluded that every identified region is indeed associated with the symptom but rather that there is a region within the cluster that shows the association (92). Finally, we would like to point out that the results reported here are of exploratory nature because this data set has been analyzed before with a mass univariate VLSM as initially intended (10).

To summarize, based on multivariate analyses of lesion location and indirect measures of disconnection, our results extend a previous study with this data set and partly confirm similar recent studies on the role of lesion locations in PSDS. Specifically, we identified an association of lesions in the right insular cortex, right putamen and inferior frontal gyrus with PSDS. Furthermore, structural disconnection in the white matter of the right temporal lobe was associated with PSDS, but there was no evidence for a contribution of functional diaschisis to PSDS. These analyses show the potential of indirect measures of disconnection and diaschisis for structure–function inference. Still, even larger cohorts, which include patients with aphasia and a more detailed behavioral characterization, are needed to leverage the full potential of LNSM and SDSM for our understanding of PSDS.

## 5. Resource identification initiative

SPM: RRID:SCR_007037; MATLAB: RRID:SCR_001622; SPSS: RRID:SCR_019096.

## Data availability statement

The raw data supporting the conclusions of this article will be made available by the authors, without undue reservation.

## Ethics statement

The studies involving human participants were reviewed and approved by the ethics committee of the Medical Faculty of Leipzig University. The patients provided their written informed consent to participate in this study.

## Author contributions

JK, DS, and MW conceptualized the study. PB and M-LB had major role in patient acquisition. K-TH had major role in acquisition of neuroimaging data. JK and MW had major role in preprocessing of neuroimaging data, analyzed the data, and performed all statistical analyses. JK drafted the manuscript. All authors contributed to the article and approved the submitted version.

## Funding

The work on this study was funded by the German Research Foundation (DFG Grant SA 1723/5-1 to DS and JK) and the Medical Faculty of the University of Leipzig (Grant 990101-113 to M-LB, Clinician Scientist Program to MW). Part of the study cohort was recruited with financial support by Merz Pharma GmbH & Co. KGaA (Frankfurt, Germany; Grant BGAAF-0136 to PB) and IPSEN Pharma GmbH (Munich, Germany; Grant BGAAF-0135 to PB). The funders were not involved in the study design, collection, analysis, interpretation of data, the writing of this article, or the decision to submit it for publication. The open access publication in Frontiers in Neurology was funded in part by the Open Access Publishing Fund of Leipzig University supported by the German Research Foundation within the program Open Access Publication Funding.

## Conflict of interest

The authors declare that the research was conducted in the absence of any commercial or financial relationships that could be construed as a potential conflict of interest.

## Publisher’s note

All claims expressed in this article are solely those of the authors and do not necessarily represent those of their affiliated organizations, or those of the publisher, the editors and the reviewers. Any product that may be evaluated in this article, or claim that may be made by its manufacturer, is not guaranteed or endorsed by the publisher.
