# Supplementary material for: Associations of lesion location, structural disconnection, and functional diaschisis with depressive symptoms post stroke
Source: Front Neurol. 2023 May 17;14:1144228. doi: 10.3389/fneur.2023.1144228 (PMC10231644; doi:10.3389/fneur.2023.1144228)
Supplement: Supplementary file 1 [file Data_Sheet_1.PDF]

Supplementary Material

Supplementary Table S1. Demographic and behavioral data.

	**All**	**PSDS-**	**PSDS+**	**p**
**Number (%)**	270	217 (80.4 %)	53 (19.6 %)	-
**Age (SD)**	66.1 (12.3)	66.1 (12.9)	66.2 (9.6)	0.97[c]
**Sex (M/F, % M)**	166/104 (62.7 %)	133/84 (63.2 %)	33/20 (60.6 %)	1.00[a]
**Stroke type (ischemic/hemorrhagic, % ischemic)**	250/20 (92.6 %)	201/16 (92.6 %)	49/4 (92.5 %)	1.00[a]
**Lesion volume in ml, mean (SD)**	18.3 (38.4)	14.5 (25.4)	34.0 (68.4)	< **0.01**[c]
**Image acquisition in days post-stroke, median (IQR)**	5 (4)	5 (3)	6 (4)	0.22[a]
**t1 in days post-stroke, mean (SD)**	6.1 (3.5)	6.1 (3.7)	6.0 (2.8)	0.57[b]
**t2 in days post-stroke, mean (SD)**	189.7 (10.2)	189.7 (10.9)	189.6 (7.0)	0.76[b]
**Barthel-Index(t1), mean (SD)**	80.6 (23.6)	82.0 (22.0)	75.0 (28.8)	0.06[b]
**NIHSS(t1), mean (SD)**	2.5 (3.3)	2.2 (3.0)	3.5 (4.3)	**0.02**[b]
**HADS-D(t1), mean (SD)**	5.0 (3.9)	4.3 (3.5)	7.9 (4.0)	**< 0.01**[b]
**HADS-D(t2), mean (SD)**	4.4 (3.7)	2.9 (2.2)	10.5 (2.2)	**< 0.01**[b]

IQR = interquartile range; SD = standard deviation; t1 = first examination; t2 = second examination; a = $\chi 2$ test; b = Mann-Whitney U test; c = two-sample t-test.

Supplementary Table S2. Anatomical labels for significant clusters.

Analyses	p-value	size in ml	Labels (% of cluster)
**SVR-LSM**<br>HADS-D continuous score<br>Cluster-level p(FWE) < 0.05	0.0096	12.488	<u>LONI</u>: unknown (30.0 %), R_insular_cortex (22.0 %), R_putamen (18.1 %), R_inferior_frontal_gyrus (14.2 %), R_superior_temporal_gyrus (4.7 %), R_caudate (2.6 %), R_lateral_orbitofrontal_gyrus (2.6 %), R_hippocampus (1.3 %), R_middle_temporal_gyrus (1.3 %), R_inferior_temporal_gyrus (1.0 %), R_postcentral_gyrus (1.0 %), R_precentral_gyrus (0.8 %), R_middle_frontal_gyrus (0.3 %), R_supramarginal_gyrus (0.3 %)<br><u>XTRACT</u>: unknown (42.9 %), Corticospinal_Tract_R (19.9 %), Superior_Thalamic_Radiation_R (12.7 %), Inferior_Fronto-Occipital_Fasciculus_R (5.2 %), Superior_Longitudinal_Fasciculus_3_R (4.9 %), Uncinate_Fasciculus_R (4.7 %), Arcuate_Fasciculus_R (3.6 %), Middle_Longitudinal_Fasciculus_R (2.6 %), Anterior_Thalamic_Radiation_R (2.3 %), Fornix_R (0.5 %), Acoustic_Radiation_R (0.3 %), Cingulum_subsection:_Temporal_R (0.3 %), Frontal_Aslant_Tract_R (0.3 %)
**SVR-LSM**<br>HADS-D continuous score with covariates (NIHSS, BI, Age, Sex)<br>Cluster-level p(FWE) < 0.05	0.0226	6.416	<u>LONI</u>: unknown (35.3 %), R_putamen (28.9 %), R_inferior_frontal_gyrus (18.4 %), R_insular_cortex (9.0 %), R_caudate (6.5 %), R_postcentral_gyrus (1.5 %), R_middle_frontal_gyrus (0.5 %)
**SVR-SDSM**<br>HADS-D continuous<br>Cluster-level p(FWE) < 0.05	0.0238	5.864	<u>XTRACT</u>: Inferior_Longitudinal_Fasciculus_R (37.7 %), unknown (26.3 %), Middle_Longitudinal_Fasciculus_R (20.6 %), Uncinate_Fasciculus_R (8.6 %), Arcuate_Fasciculus_R (5.1 %), Fornix_R (1.7 %)

Anatomical labels were calculated for all significant clusters from the SVR-LSM and SVR-SDSM (p(FWE) < 0.05) based on the LONI probabilistic atlas for gray matter and the XTRACT atlas for white matter.

Supplementary Table S3. SVR-LSM and SVR-SDSM parameters.

scores	hyperparameters	Correlation of predicted vs. real scores for full model
SVR-LSM, corrected for lesion size	C = 30.00, kernel = 0.45, ε = 0.10	r = 0.930, rank 1/5000, p < 0.001
SVR-LSM, corrected for lesion size, age, sex, stroke severity (NIHSS) and functional impairment (Barthel-Index)	C = 30.00, kernel = 0.45, ε = 0.10	r = 0.906, rank 1/5000, p < 0.001
SVR-SDSM, corrected for lesion size	C = 30.00, kernel = 0.45, ε = 0.10	r = 0.928, rank 1/5000, p < 0.001

C = Cost/Box constraint
